# Fasting Mimicking Diet Cycles, Regeneration, Biological Age, and Disease

**DOI:** 10.1093/geroni/igaf122.1389

**Published:** 2025-12-31

**Authors:** Valter Longo

**Affiliations:** USC Leonard Davis School of Gerontology, Los Angeles, California, United States

## Abstract

Fasting mimicking diets (FMDs) are low calorie and protein and high fat compositions lasting 4-7 days emerging as periodic dietary interventions with the potential to improve healthspan and decrease the incidence of age-related diseases. In animal studies, FMD cycles promote stem cell activation, cellular reprogramming and autophagy leading to longevity extension but also to the regression of multiple diseases and conditions. Recent randomized clinical studies consistently indicate that FMD cycles reverse insulin resistance and promote pre-diabetes and diabetes regression and result in lower diabetes and hypertension drug use by a mechanisms not correlated with weight loss. The latest studies indicate that FMD cycles can improve clinical response to autoimmune disease drugs and reverse chemosensory dysfunction raising the possibility that the multi-system regenerative effects demonstrated in mice and rats are conserved in humans, as also supported by a 2.5 years of biological age reduction associated with 3 FMD cycles in 2 different clinical trials.

